# Supplementary material for: Role and mechanism of NCAPD3 in promoting malignant behaviors in gastric cancer
Source: Front Pharmacol. 2024 Apr 22;15:1341039. doi: 10.3389/fphar.2024.1341039 (PMC11070777; doi:10.3389/fphar.2024.1341039)
Supplement: Supplementary file 11 [file DataSheet2.ZIP › GSEA/immunologic/my_analysis.Gsea.1599462003817/GSE6259_DEC205_POS_DC_VS_CD4_TCELL_DN.html]

Details for gene set GSE6259\_DEC205\_POS\_DC\_VS\_CD4\_TCELL\_DN[GSEA]

|  || Dataset | filtered\_dataset.sample\_info.cls#WT\_versus\_NCAPD3\_MUT |
| Phenotype | sample\_info.cls#WT\_versus\_NCAPD3\_MUT |
| Upregulated in class | WT |
| GeneSet | GSE6259\_DEC205\_POS\_DC\_VS\_CD4\_TCELL\_DN |
| Enrichment Score (ES) | 0.48170665 |
| Normalized Enrichment Score (NES) | 1.9091254 |
| Nominal p-value | 0.0017452007 |
| FDR q-value | 0.57089657 |
| FWER p-Value | 1.0 |
Table: GSEA Results Summary

  

Fig 1: Enrichment plot: GSE6259\_DEC205\_POS\_DC\_VS\_CD4\_TCELL\_DN      
 Profile of the Running ES Score & Positions of GeneSet Members on the Rank Ordered List

  

| SYMBOL | TITLE | RANK IN GENE LIST | RANK METRIC SCORE | RUNNING ES | CORE ENRICHMENT || 1 | 5087 | PBX1 | 54 | 0.908 | 0.0634 | Yes |
| 2 | 10207 | INADL | 73 | 0.851 | 0.1462 | Yes |
| 3 | 26278 | SACS | 205 | 0.669 | 0.1275 | Yes |
| 4 | 54865 | GPATCH4 | 261 | 0.622 | 0.1580 | Yes |
| 5 | 5810 | RAD1 | 360 | 0.560 | 0.1507 | Yes |
| 6 | 57109 | REXO4 | 370 | 0.552 | 0.2063 | Yes |
| 7 | 1387 | CREBBP | 387 | 0.544 | 0.2560 | Yes |
| 8 | 23567 | ZNF346 | 393 | 0.541 | 0.3133 | Yes |
| 9 | 7334 | UBE2N | 546 | 0.449 | 0.2547 | Yes |
| 10 | 55339 | WDR33 | 551 | 0.445 | 0.3019 | Yes |
| 11 | 79080 | CCDC86 | 555 | 0.443 | 0.3496 | Yes |
| 12 | 27349 | MCAT | 574 | 0.436 | 0.3857 | Yes |
| 13 | 9807 | IP6K1 | 605 | 0.420 | 0.4115 | Yes |
| 14 | 54512 | EXOSC4 | 623 | 0.411 | 0.4455 | Yes |
| 15 | 286410 | ATP11C | 637 | 0.405 | 0.4817 | Yes |
| 16 | 55646 | LYAR | 761 | 0.342 | 0.4319 | No |
| 17 | 84823 | LMNB2 | 833 | 0.293 | 0.4139 | No |
Table: GSEA details [plain text format]

  

Fig 2: GSE6259\_DEC205\_POS\_DC\_VS\_CD4\_TCELL\_DN      
 Blue-Pink O' Gram in the Space of the Analyzed GeneSet

  

Fig 3: GSE6259\_DEC205\_POS\_DC\_VS\_CD4\_TCELL\_DN: Random ES distribution      
 Gene set null distribution of ES for **GSE6259\_DEC205\_POS\_DC\_VS\_CD4\_TCELL\_DN**
